# The Role of Endometrial Microbiota in Fertility and Reproductive Health: A Narrative Review

**DOI:** 10.7759/cureus.78982

**Published:** 2025-02-14

**Authors:** Priti Karadbhajne, Akash More, Hellen Y Dzoagbe

**Affiliations:** 1 Clinical Embryology, Datta Meghe Institute of Higher Education and Research, Wardha, IND; 2 Obstetrics and Gynecology, Acharya Vinoba Bhave Rural Hospital, Wardha, IND

**Keywords:** dysbiosis, endometrial infection, hormonal changes, infertility, lactobacillus

## Abstract

This review explores the correlation between endometrial microbes and infertility, focusing on the impact of endometrial microbiota on fertility outcomes. The healthy status of uterine microorganisms, predominantly *Lactobacillus*, plays a crucial role in reproductive health. Studies have shown that the endometrial microbiota composition changes with hormonal fluctuations during the menstrual cycle, affecting endometrial receptivity and embryo implantation. The presence of specific bacteria, such as *Lactobacillus *species, has been associated with successful pregnancy outcomes, while dysbiosis in the endometrial microbiota has been linked to implantation failure and infertility. Factors like age, hormonal changes, and immune responses influence the composition of endometrial microbes, impacting fertility. Chronic endometritis, characterized by inflammation of the endometrium, has been linked to recurrent implantation failure and infertility. Understanding the complex interactions between endometrial microbiota, immune responses, and hormonal influences is crucial for improving fertility treatments and outcomes.

## Introduction and background

A description of the healthy status of uterine microorganisms at the stage of reproduction in females is given by most of the studies that show the dominance of a single microbe, *Lactobacillus *[1]. As holobionts, humans and a variety of microbes, such as bacteria, viruses, fungi, yeast, and archaea, have developed together [2]. These microorganisms and the genetic material they contain, known as the microbiome, are now thoroughly studied; the Human Microbiome Project has shown that the female reproductive tract contains around 9% of the entire human microbiome. In the past, it was thought that only the lower part of the genital canal was home to bacteria, and the cervix served as an ideal barrier to keep the uterus sterile by separating the vagina from the upper genital tract [3]. Successful embryo implantation requires synchrony of the development of a grade embryo and a healthy endometrium. Poor embryo grade is responsible for nearly one-third of the in vitro fertilization (IVF) population implantation failure, and the remaining failure accounts for endometrial receptivity [3,4].

Conception failure is linked in many infertile women with assisted reproductive technology (ART) with multiple IVF cycles. Recent studies have highlighted the importance of assessing microbes in the endometrium for embryo development, embryo attachment/apposition/invasion, and early pregnancy progression. Few studies analyzed if endometrial microbiota can influence the prenatal conditions of the mother and subsequently affect endometrial receptivity [5,6]. The microbiota, cellular immune response, and cytokines maintain the stability of the endometrium, and changes in these factors affect its receptivity. Embryo attachment in the epithelium wall of the endometrium is regulated with an equilibrium of inflammatory factors [3].

The female reproductive tract comprises 9-10% of the population of the whole human microbiome. From the vagina to the ovaries, bacterial diversity differs, i.e., outer to inner organs and bacterial diversity decreases. The presence of a variety of microbes in both male and female reproductive organs affects reproductive function. A 16S rRNA reveals that there is an impact on infertility in patients [3,4]. The endometrial microbiota can also be affected by hormonal changes; a small study indicated that by using gonadotropin-releasing hormone agonist (GnRHa) for patients undergoing endometriosis treatment, a significant decrease in *Lactobacillus *with an increase in other bacterial populations was reported [5]. Successful embryo implantation requires synchrony of the development of a grade embryo and a healthy endometrium. Poor embryo grade is responsible for nearly one-third of IVF population implantation failure, and the remaining failure accounts for endometrial receptivity [4]. This review explains the relationship between endometrial infection and infertility.

## Review

Search methodology

To find pertinent material for this comprehensive review, a thorough search approach was carefully carried out. The principal objective was to provide a thorough narrative of microbial dynamics in the female reproductive tract and to provide insights into their role in endometrial health and infertility. A thorough probe was carried out across respectable academic resources, including PubMed, Web of Science, Scopus, and Google Scholar, covering a significant number of current research findings published in studies between 2014 and the present. The databases were searched using precise, topic-specific keywords to guarantee the inclusion of relevant papers. Keywords such as "infertility," "dysbiosis," "lactobacillus," "endometrial infection," and "hormonal changes" were purposefully employed. The thorough retrieval of papers that emphasized microbial dynamics and their effect on endometrial health and infertility was made possible by this methodical keyword approach, ensuring a complete and up-to-date analysis.

Carefully chosen articles, reviews, and reports were selected based on predetermined inclusion and exclusion standards that were customized for the subject matter. The chosen sources had to meet two requirements for inclusion: they had to be in English and discuss current events in microbial dynamics and their effect on endometrial health and infertility. We made sure that the analysis was founded on the most recent and pertinent research in the field by concentrating on studies done between 2014 and 2024. The purpose of this stringent selection procedure was to retrieve and scrutinize the current and extensive body of literature that provides insights into how the complex mechanics of endometrial microbiota affect endometrial health and infertility.

Endometrium

To know the importance of microbes in the female reproductive tract, there is a need to comprehend how the endometrium serves as the foundation for an effective implant and placentation. Clinicians and scientists are keen to understand the embryo implantation process because there is very little available information about the mechanism of the implantation process. Every month, the stage for placenta creation is set prior to the appearance of a developing embryo. The transformation of the endometrium into a receptive state throughout the mid-to-late luteal phase is not dependent on the existence or absence of a conceptus. The receptive ability of the endometrium is between 19 to 24 days of menstruation; this is considered a window of implantation, enduring for two to four days. Variations in progesterone and estrogen coordinate the significant hormonal changes required for proper cyclical alterations of the endometrium [4]. The proliferation of columnar epithelial cells to glandular cells in between the secretory phase of the menstrual cycle stages and the formation of a robust barrier through strong junctions take place at the uterus [5]. Antimicrobial peptides (AMPs), which are responsible for preventing or suppressing infection, are present in both the endometrial fluid (EF) and the uterine mucosal surface. The concentration of AMPs varies throughout the menstrual cycle [3]. It is well-recognized that AMPs support the health of the female reproductive system, which has an impact on pregnancy and fertility. The secretory leukocyte protease inhibitor, which has antiviral and antifungal qualities and functions as a bactericidal against both gram-positive and gram-negative bacteria, including *Staphylococcus aureus* and *Escherichia coli*, is an example of an AMP discovered in the uterus [5].

The Framework and Origin of Endometrial Microorganisms in Healthy Women

Functional and compositional aspects of endometrial microorganisms endure debate. The general opinion for the healthy endometrium is the presence of colonization of *Lactobacillus *mostly. However, some studies revealed different observations. According to Verstraelen et al., most of the studied endometrial bacterial profiles revealed three common Bacteroides and Pseudomonas species [6]. Another study found that Acinetobacter, Pseudomonas, Comamonadaceae, and Cloacibacterium control the outline of endometrial microbes after a hysterectomy-like procedure. Chen et al. also proved the same [7]. Five general categories have been discovered for the vaginal microbiome, of which four have been found to be dominated by* Lactobacillus*: Group V (5.3%) is dominated by *L. jensenii; *Group III (34.1%) is dominated by* L. iners*; Group II (6.3%) is dominated by *L. gasseri*; and Group I (26.2% of the sampled population) is dominated by *Lactobacillus crispatus*. Group IV has, however, been classified as non-*Lactobacillus *dominated, which includes *Gardnerella *and Pretovell [5,8].

**Figure 1:**
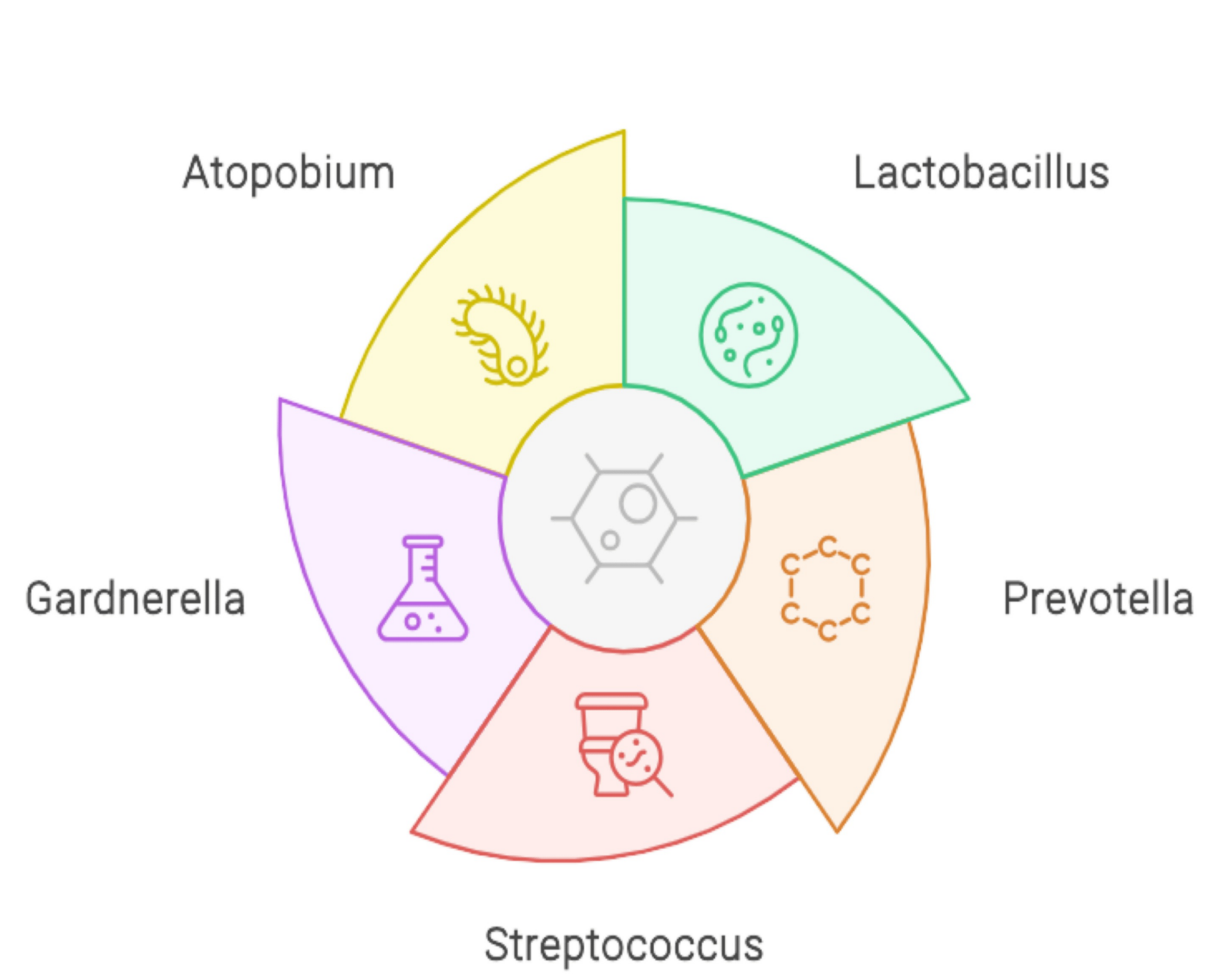
Composition of endometrial microbiota Image Credits: Priti Karadbhajne and Hellen Y. Dzoagbe created this figure using the Canva platform.

The high estrogen and progesterone levels cause *Lactobacillus *dominance; estrogen causes glycogen deposition in the epithelium of the vagina, and progesterone conducts cytolysis of cells that releases glycogen and gets metabolized to lactic acid by bacillus, giving the pH range of the vagina to be around 4 to 5. Alteration in the endometrial microbial profile is observed due to progesterone and estradiol hormones at different stages of the menstrual cycle [9]. Chen et al. also observed variation in endometrial microbes in their study during the proliferative and secretory phases of the menstrual cycle [10]. The placental and endometrial microbiomes are independent as the placental development originates from decidualized endometrium, and mainly, the trophoblast cells should be in contact with the residential microbiota. Also, placental colonization takes place from the bacteria previously residing in the uterus, and also vaginal origin is responsible for uterine colonization with the population residing in both sites of the organ [11]. Figure 1 depicts the composition of endometrial microbiota.

Obstacles to Exploration of Endometrial Microbes

It is quite likely that the endometrial microbiota influences reproductive health, which in turn influences the course of pregnancy. Whole genome sequencing, targeted sequencing, DNA microarrays, 16S rRNA sequencing, and DNA fingerprinting are examples of molecular methods and metagenomics. Certain molecular techniques are employed to identify bacteria for which in vitro culture is difficult. The 16S RNA sequence-generated operational taxonomic units (OTUs) indicate the existence or lack of genetic material for further investigation, but they do not reflect the survival of the bacteria that have been detected. Also, quantification of a particular species/organism is a challenge when it comes to newly identified ones. It even deprives one of knowledge of the biological function, providing only physiological and ecological approaches [10].

Studies assessing the clinical influence of the microbiome on the efficacy of ART have been conducted as the microbiota of the female reproductive tract has undergone thorough investigation and has been precisely characterized. Such work could shed light on unsuccessful treatments that were hitherto unclear. Elnashar estimated in a study that the vagina may be the habitat of nearly about 1010-1011 bacterial species, and the endometrium may have fewer bacteria than the vagina. The study of the endometrium is interrupted due to contaminations secreted by bacteria, lab equipment, and reagents during sample collection and the presence of DNA bacteria in the air [12].

Association Between Endometrial Microbiota and Receptive Ability of Endometrium and Implantation

Embryo attachment, invasion, and development are complex processes known as the receptivity of the endometrium. There is a short period of 3-4 days that is suitable for embryo implantation in the endometrium; it is known as a window of implantation. The implantation window appears after ovulation on days six to eight. Normally, failure of implantation occurs because of the low quality of the embryo and endometrium. Endometrium quality is measured in terms of thickness, vascularity, and good physical condition (without infection). Some factors are known for the obstruction of the endometrial receptivity, such as endocrine, inflammation, thin endometrium, granuloma, tumor, septum, and immunity-related disorders. Endometrial receptivity array (ERA) is one of the methods used for accurate and frequent identification of the implantation window for implantation [4]. In the endometrial cycle, during the proliferative phase, the thickness of the endometrium increases with estrogen levels, and progesterone increases in a secretory phase that makes the endometrium easy to implant and ready for receiving an embryo. Asynchrony between the embryo and the endometrium increases the risk of miscarriage during this time. Studies suggest that mainly the secretory phase undergoes major characteristic changes and helps in pregnancy [4,5,8,12].

**Figure 2:**
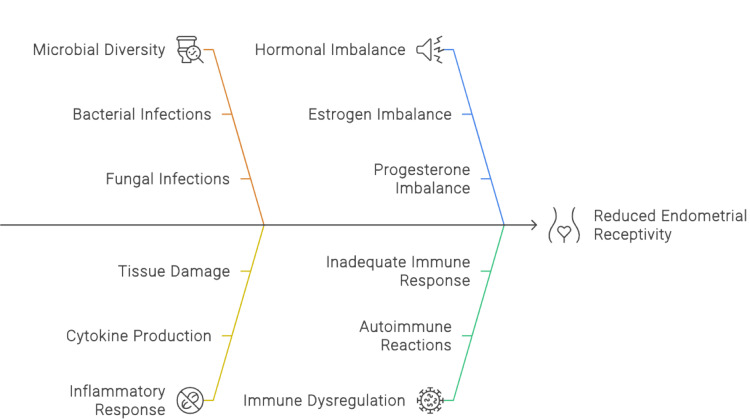
Association between endometrial microbes and receptivity Image Credits: Priti Karadbhajne and Hellen Y. Dzoagbe created this figure using the Canva platform.

In the implantation process, the apposition of the blastocyst to the endometrium, as well as the attachment and invasion of the trophectoderm to the endometrium, usually takes place. Mucin is MUC-1 hormone-modulated and associated with the nonreceptive endometrium. Women with abnormal fertility show higher MUC1 expression [5]. When the zona pellucida gets removed from the embryo, adhesion and attachment show a stronger physical bond so that the embryo cannot be dislodged from the endometrial surface. Adhesion is mediated by integrins, cadherins, selectins, and immunoglobulins [13]. When comparing the infertile group to the fertile group, *Lactobacillus *​​​​​​​*gasseri* is highly prevalent, whereas *Lactobacillus *​​​​​​*iners *and *L. crispatus* are lacking. *Staphylococcus pasteuri/warner *and* villanelle* are more prevalent in the study that looks into the vaginal microbiota of women receiving ART. They discovered that *Lactobacillus *predominated in the vaginal swabs of every other woman - with the exception of one woman - who had delivered live babies. Figure 2 shows the association between endometrial microbes and fertility.

Local Immunity Modulation of the Endometrium

Recent research proved that the function and phenotype of immune cells are varied by microorganisms. The relationship between local immunity and microorganisms remains always debatable. According to Wang's hypothesis, the microorganisms may prove harmless and similar in many patients having healthy endometrium, and it is always examined by the immune regulatory system. The inflammatory environment is balanced by the mutual connection between endometrial microbes and the local immune system [14,15]. Various immune cells play key roles in immunity processes related to the uterus, such as natural killer (NK) cells, macrophages, T cells, neutrophils, and mast cells [5,15]. In the female reproductive tract, there is the presence of a physical barrier against microbes known as the first line of defense. Pathogen colonization resistance is the result of immune defense against the exterior environment done by the mucosal barriers, IgA antibodies, and common microbes present in the female reproductive tract [14].

The uterine microbiota and its immune system have to undergo adaptations during the menstrual cycle, implantation, and pregnancy. The paternal antigens created as an immunological stimulation by the semen, embryo, and fetus are overcome together by uterine microbiota and immune cells again [5]. Endometrial immune profile (EIP) is a test to diagnose the molecular immune profile of the lining of the uterus (endometrium) to observe that it is ready to resume a positive pregnancy. Changes in local microbial communities can lead to reproductive failure, i.e., from implantation failure to complications in pregnancy and other gynecological disorders/diseases. Tumorigenesis is a major alteration of uterine microbiota. The presence of *Atopobium vaginae *and *Porphyromonas spp*. in the female reproductive tract resumes endometrial cancer like other microbiota responsible for particular organ cancers in the human body generally [4,5]. Few consider it as a chronic inflammatory process associated with immune processes. Macrophages recognize the foreign cells and present them to T cells. These NK cells are responsible for the phenomenon of natural cytotoxicity in the immune system. Lesser functioning of NK cells reduces their ability to purge the peritoneal cavity of endometrial elements after a reverse outflow of menstrual blood [8]. The uterine microbiota, along with innate and adaptive immunity, has a symbiotic relationship in maintaining an inflammatory response as this bacterial stimulation induces a favorable environment for embryo implantation. On the other hand, a regulated stimulation can give rise to tolerant non-sterile transfer of semen through the uterine cavity [4,5].

Role of Hormones Involved in Reproduction

Variation in the microorganisms of the endometrium is observed at various phases of the menstrual cycle and may be due to the modulation of hormones on endometrial immune status. The release of pro-inflammatory cytokines, chemokines, and AMPs is influenced by sex hormones during the female reproductive period; these hormones also play a role in the selection of vaginal microbial species. Estradiol hormone causes glycogen synthesis and the generation of maltose, maltotriose, and α-dextrins in epithelial cells; these are used as a substrate in the process of turning lactic acid by lactobacilli. This is the reason for the adaptation of the microorganisms of the vagina from a *Lactobacillus *deficiency to a proliferative *Lactobacillus *state [5]. Hormonal variations, particularly those related to estrogens, have been linked to regulating the vaginal microbiota as well as the endometrial preparation for embryo transfer success and pregnancy [12]. According to Moreno, endometrial microbes do not change with hormones before the time period of implantation [3].

Endometrial Microbes and Infertility

Endometrial microbiota composition in patients receiving ART was examined by analyzing EF and endometrial biopsy using 16s rRNA sequencing. This analysis showed that while a high count of *Lactobacillus *contributed to achieving pregnancy, the existence of *Gardnerella, Klebsiella, Hemophilus, Staphylococcus, Neisseria, Bifidobacterium, Streptococcus, Atropobium, *and *Chryseobacterium* was linked to clinical miscarriage and no pregnancy. One can say that reduction in *Lactobacillus spp.*(vaginal homeostasis) is parallel to increased bacterial colonization giving diverse microbiota that leads to gynecological problems [5].

Effect of Endometrial Microbes on Endometrial Receptivity

It is currently unknown how the disruption of the endometrial microbes affects the implantation process adversely. A disrupted equilibrium is consistent with the identified mechanism of the link between endometrial cells and the microbiota. This may occur with the loosening of the epithelial barrier as a result of the non-commensal microbiome's predominance, which restricts the host's defenses and makes it easier for germs to access the endometrial stroma [4].

Endometritis

The existence of the endometrium outside the cavity of the uterus combined with persistent inflammation is thought to be endometritis. In his work "Disputatio Inauguralis Medica de Ulceribus Ulceri," Daniel Shroen initially reported this ailment in 1690. Arthur Duff followed up with the disease's symptoms in 1769. It is distinguished by the existence of functional foci of endometrial tissue (endometrioides; Gr. eides-similar) or glandular cells and stroma of the endometrium outside of its cavity, primarily in the uterine muscle layer, other genitals and their environs, and even in locations remote from the human body's genital organs [16]. It can manifest outside of the uterus, in the bladder, ureters, ovaries, or peritoneal cavity. It is an estrogen-dependent, benign gynecological illness [17]. Endometritis isn't a life-threatening condition, but it can cause complications. Mainly, endometritis is a result of uterine infection, which is long-term or short-lived. Acute endometritis is not dangerous as the right dosage of antibiotics helps in the treatment and hence returning back to a normal lifestyle. Acute endometritis is mainly the neutrophil invasion with micro abscess formation in the superficial epithelium, gland lumina, and uterine cavity. However, this kind of endometritis is not associated with infertility or fewer chances of pregnancy [11,17]. In chronic endometritis (CE), there is the presence of a high number of stromal cells, as well as infiltration of endometrial stromal plasma cells (EPSCs). Specifically, multiple EPSCs are considered a good pathological indication [11].

Chronic Endometritis

CE is associated with frequent miscarriages. In this, embryos cannot be implanted or grown when the lining of the uterus is irritated and inflamed. This condition is also termed recurrent/repeated implantation failure (RIF). The long-term impact of CE makes it challenging to get pregnant [16]. Inflammation in the mucosa with the presence of edema, high stomal density, and the presence of cell infiltrate in the stroma is a characterized chronic type. A proposed model for chronic endometriosis is one in which a microbial infection of the cavity induces the expression of inflammatory molecules that trigger unusual immune responses in the endometrium. The B cells circulate in the stoma compartment and further go into glandular areas. Likewise, endometrial B cells differentiate into EPSCs. This results in an increase in the presence of antibodies in the mucosa, which negatively impacts the embryo implantation process [18].

Microorganisms in Chronic Endometritis

The primary source of CE is infection in the uterine cavity. Typically, this infection is caused by mycoplasma/ureaplasma species, such as *Mycoplasma genitalium*, *M. hominis*, and *Ureaplasma urealyticum*; Proteus species, comprising bacteria like *E. coli,* S*taphylococcus, Streptococcus *species, and* Enterococcus faecalis* species; and finally, primarily two yeast species, candida species and *Saccharomyces cerevisiae*. *Mycobacterium tuberculosis* is sometimes identified as the causative agent of chronic granulomatous endometritis, which is regarded as a type of CE.* Chlamydia trachomatis* and *Neisseria gonorrhoeae* are other microbes that take part in CE, but their relationship remains undetermined [19-21]. Few cases expose the altered level, i.e., an imbalance in Lactobacilli population species in the female tract mainly increases in lactobacilli, which is a proven observation in CE for a few patients. If closely observed, then the microorganisms in endometrial tissue that are inconsistent are different from those in the vaginal discharge. This suggests that sampling using the lower genital tract is unpredictable [22].

Inflammation in Chronic Endometritis

The percentage of B lymphocytes in the total leukocyte population in the healthy human endometrium is below 1%. As core cells in distinct lymphocyte aggregates encircled by a profusion of CD8(+) T cells and macrophages, endometrial B cells are primarily observed in the basal layer (the region that endures throughout the monthly cycle) [7]. It is still unclear what function B cells and lymphocyte aggregates serve in the human endometrium [13,17,22]. In the basal layer, endometrial B cells are encircled by a large number of macrophages and CD8+ T cells. The basal layer and the functional layer of CE are where B cells penetrate. The B cells reach gland lumina through the stomal compartment via epithelial areas. In the secretory phase, a lower percentage of CD16-negative, CD56-positive/bright, and NK cells are observed, compared with those without CE, along with an increase in T cells, which indicates aberrant mucosal composition [7,23].

Chemokines and adhesion molecules like CD62E, CXCL1, and CXCL13 are expressed in epithelial cells in CE; even interleukin IL-6 has a higher side presence in menstrual effluents in patients with CE. Lipopolysaccharides are responsible for the induction of pro-inflammatory molecules via microbial infection. Hence, there is a circulation of B-cells in the stroma and glandular areas, and some differentiate into ESPCs [17]. The IL-1β and tumor necrosis factor (TNF)-α levels are also elevated in CE, which raises the estrogen biosynthesis in glandular cells of the endometrium, giving rise to endometrial micropolyposis, finding that it is often seen in CE through hysteroscopy. A high level of immunoglobulin (Ig) subclasses (IgM, IgA1, IgA2, IgG1, and IgG2) with a predominance of IgG2 is observed due to EPSCs in CE. Due to such high expression, there is a negative impact on embryo implantation [17].

Due to differentiation in the mid-secretory phase in infertile women with CE, pseudostratification and mitotic nuclei in glandular and epithelial cells are seen in the secretory phase of the disease. In contrast, ovarian steroid receptors (progesterone receptors A and B, estrogen receptors α and β, and decidualization genes prolactin (PRL) and insulin-like growth factor binding protein 1 (IGFBP1) that are linked to embryo receptivity, are downregulated during the secretory phase of CE. A small number of antiapoptotic genes - B-cell lymphoma 2 (BCL2) and BCL2-associated X (BAX), proliferation-associated nuclear marker (Ki-67), and ovarian steroid receptors - are upregulated. So ultimately, endometrium with CE is unresponsive to ovarian steroids, resulting in progesterone resistance, as also in endometriosis [7].

Previously, CE was not in the limelight in the field of gynecology, as often patients suffering were asymptomatic and not pathologically specific overall. But later, its diagnosis became essential as it has been closely related to unexplained fertility. Hysteroscopy of the uterine cavity and endometrial biopsy with the identification of plasma cells histologically are the keen ways to diagnose endometritis. Diagnostic accuracy is modest in hysteroscopy. Immunological detection of transmembrane heparan sulfate proteoglycan syndecan-1 (CD138), a specific marker of plasma cells, has a good accuracy rate in diagnosis, and on the other hand, microbiological cultures are used as direct identification for appropriate antibiotic treatment [7].

Additionally, molecular techniques like the next-generation sequencing (NGS) of 16S ribosomal subunits and reverse transcription polymerase chain reaction (RT-PCR) are a gateway to identifying pathology and endometritis. Though there is an increased interest in this field, no specific study has been published yet.

Recommendations and future direction

To improve our understanding of the role of endometrial microbiota in reproductive health, future research should focus on adopting advanced molecular techniques, such as NGC and RT-PCR, for precise identification and quantification of microbial communities. Standardizing protocols for sample collection, processing, and analysis is crucial to minimize contamination and ensure the reliability of results. Longitudinal studies are needed to investigate how fluctuations in endometrial microbiota throughout the menstrual cycle influence endometrial receptivity and implantation success. Additionally, clinical trials should evaluate the effects of probiotic and prebiotic interventions on endometrial microbiota composition and reproductive outcomes. These approaches will help in developing targeted therapeutic strategies to improve fertility, address recurrent implantation failures, and enhance our understanding of microbial dynamics in the female reproductive tract. Such advancements could lead to more effective treatments and better management of infertility and related reproductive health issues.

## Conclusions

The intricate relationship between endometrial microbiota and fertility outcomes underscores the importance of considering microbial factors in reproductive health. Studies have highlighted the significant impact of menstrual cycle-related changes in the endometrial microbiota on IVF success rates and implantation outcomes. Dysbiosis in the endometrial microbiota can lead to infertility issues and recurrent implantation failure. Assessing the composition of endometrial microbes may provide valuable insights into predicting reproductive success in ARTs. Further research is needed to elucidate the mechanisms by which endometrial microbiota influence fertility outcomes. It is also recommended that clinicians consider assessing endometrial microbiota as part of routine fertility evaluations to optimize treatment strategies. Developing personalized interventions targeting the endometrial microbiota could potentially improve IVF success rates. However, longitudinal studies investigating the impact of hormonal fluctuations on endometrial microbial composition are warranted, which calls for collaboration between researchers, clinicians, and microbiologists to advance our understanding of how endometrial microbes affect fertility and pregnancy outcomes.
